# Late-Presenting Congenital Subaortic Membrane With Aortic Regurgitation Presenting as Dyspnoea: A Case Report and Review of the Literature

**DOI:** 10.7759/cureus.92876

**Published:** 2025-09-21

**Authors:** Ahmed Abouelazm, Kirollos Philops

**Affiliations:** 1 Cardiology, Portsmouth University Hospital, Portsmouth, GBR

**Keywords:** aortic regurgitation, cardiac computed tomography, echocardiography, left ventricular outflow tract obstruction, subaortic membrane

## Abstract

Subaortic membrane (SAM) is a rare congenital cause of left ventricular outflow tract (LVOT) obstruction. While often diagnosed in childhood, it can remain clinically silent until adolescence or adulthood, presenting diagnostic and management challenges due to its indolent progression and lack of adult-specific guidelines. SAM is frequently associated with progressive aortic valve pathology, including aortic regurgitation (AR).

We report the case of an 18-year-old male with a previously unrecognised congenital subaortic membrane presenting with exertional dyspnoea and dizziness. Transthoracic echocardiography revealed a discrete 0.5 cm subaortic membrane, a peak left ventricular outflow tract (LVOT) gradient of 45 mmHg, and mild-to-moderate central AR. Cardiac computed tomography (CT) confirmed the lesion with no coronary anomalies. The patient underwent successful surgical resection of the membrane, resulting in a reduced post-operative LVOT gradient (peak 1.68 m/s, mean gradient (MG) 6 mmHg) and resolution of symptoms. Follow-up echocardiography at three months confirmed persistence of only mild AR with no residual LVOT gradient, and the patient reported full return to premorbid functional status (New York Heart Association (NYHA) I), highlighting favourable short-term outcomes.

This case highlights the importance of recognising late-presenting congenital SAM in symptomatic adolescents and young adults. Early diagnosis, appropriate imaging, and timely surgical intervention are essential. The case underscores the need for standardised long-term surveillance and the development of adult-specific management strategies, especially considering the high risk of recurrence after surgery.

## Introduction

Subaortic membrane (SAM) is a rare congenital cardiac anomaly characterised by a fibrous or fibromuscular ridge in the left ventricular outflow tract (LVOT), just below the aortic valve (AV). It accounts for 6-8% of adult congenital heart disease and contributes to 8-20% of LVOT obstructions in this population, though its true incidence may be underestimated due to a delayed or missed diagnosis [1,2]. While commonly diagnosed in children, adult-onset cases are increasingly recognised due to advances in imaging and clinical awareness [3]. SAM is associated with progressive left ventricular hypertrophy, and its turbulence often leads to damage of the AV and aortic regurgitation (AR), which occurs in up to 80% of untreated adult patients with SAM [4].

The precise aetiology of SAM remains unclear, but structural anomalies, such as increased mitral-aortic separation and steepened aorto-septal angles, are proposed contributors [5,6]. These factors alter blood flow trajectories during cardiac development, promoting fibrous membrane formation. Although symptoms may be subtle, ranging from exertional dyspnoea to dizziness, diagnosis in adults can be challenging, as SAM can mimic conditions like hypertrophic cardiomyopathy or rheumatic valve disease [5,7].

Transthoracic and transoesophageal echocardiography are key diagnostic tools, while cardiac computerised tomography (CT) and magnetic resonance imaging (MRI) are valuable for preoperative planning and delineating complex anatomy [3,6]. Management is largely surgical. Surgical resection is indicated in symptomatic patients or those with a peak LVOT gradient ≥50 mmHg [7]. However, postoperative recurrence rates range from 20% to 55%, and up to one-third of patients may require reoperation, particularly if initial excision is incomplete [1,7].

Importantly, adult-onset cases may differ pathophysiologically from paediatric forms, as they are thought to arise from slower fibroelastic proliferation rather than congenital embryological maldevelopment, leading to a more indolent clinical course but equally high recurrence risk [4].

Despite these risks, long-term surveillance strategies and adult-specific guidelines remain poorly defined. This case highlights a young adult patient with SAM and moderate AR, contributing to a growing need for nuanced, age-specific treatment protocols and long-term monitoring strategies [3,7].

## Case presentation

Clinical history

An 18-year-old, previously healthy male presented with a 1-year history of exertional breathlessness (NYHA Classes II-III) and occasional dizziness during physical activity, notably while playing basketball. Symptoms were transient and relieved with rest. He denied syncope, palpitations, chest pain, or orthopnoea. There was no significant family history of cardiovascular disease, and he reported no tobacco, alcohol, or illicit drug use. His body mass index (BMI) was 22.1 kg/m², with a calculated body surface area of 1.9 m².

Physical examination

At the cardiology outpatient clinic, the patient was haemodynamically stable and afebrile. His vital signs included a blood pressure of 110/70 mmHg, heart rate of 75 bpm, and oxygen saturation of 98% on room air. Physical examination revealed a harsh, grade 3/6 mid-systolic murmur best heard at the left mid-sternal border and a diastolic murmur accentuated during forward sitting with expiration. No peripheral signs of heart failure were noted.

Initial investigations

**Figure 1:**
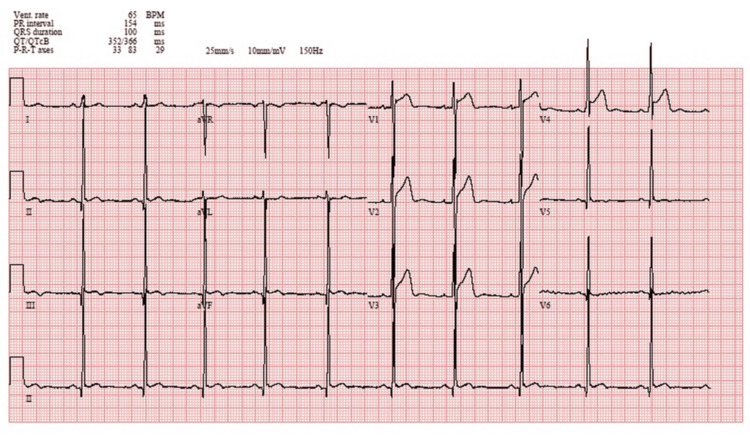
Standard 12-lead electrocardiogram Standard 12-lead electrocardiogram demonstrating normal sinus rhythm at 65 bpm. Evidence of left ventricular hypertrophy (LVH) is present based on voltage criteria (tall R waves in V5–V6 and deep S waves in V1). No ischemic changes, conduction abnormalities, or ST-T segment deviations are noted.

**Figure 2:**
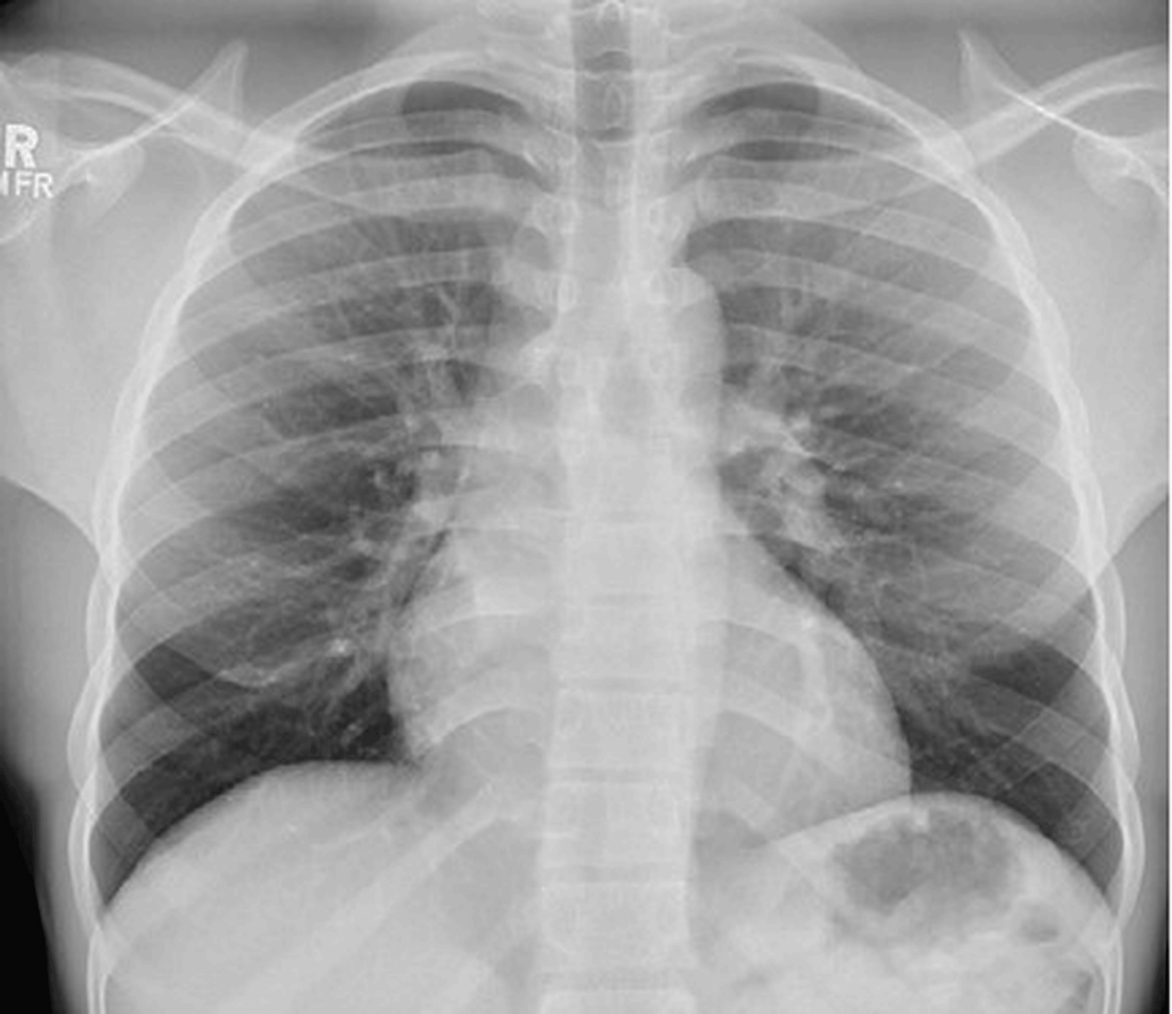
Initial chest radiograph Posteroanterior chest X-ray showing a normal cardiothoracic ratio with no signs of pulmonary congestion, cardiomegaly, or pleural effusion. Lung fields appear clear, and bony thoracic structures are unremarkable.

Initial investigations revealed a chest X-ray (Figure 1) showing a normal cardiothoracic ratio with no evidence of pulmonary congestion or other abnormalities. The electrocardiogram (ECG) (Figure 2) demonstrated sinus rhythm with features consistent with left ventricular hypertrophy (LVH).

**Figure 3:**
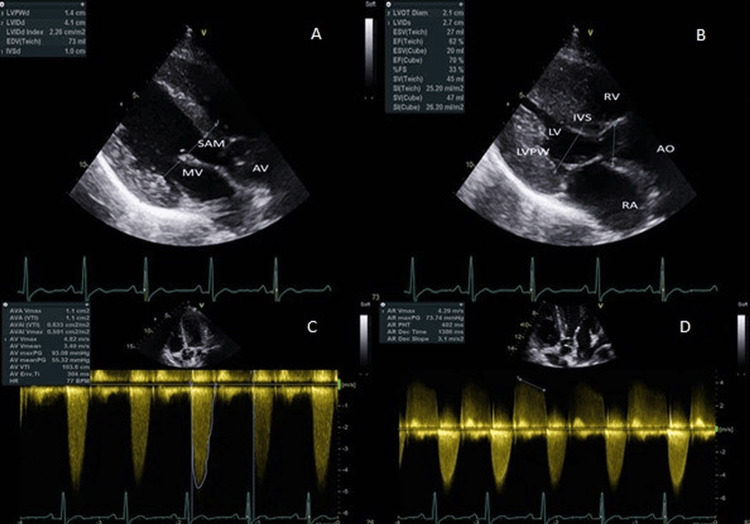
Echocardiographic imaging demonstrating subaortic membrane and hemodynamic impact Top panel: Two-dimensional transthoracic echocardiography in the parasternal long-axis view showing a discrete subaortic membrane (SAM) within the left ventricular outflow tract (LVOT) during diastole (A) and systole (B). The images also demonstrate increased diastolic wall thickness of the left ventricular posterior wall (LVPWd 1.4 cm), consistent with concentric hypertrophy, with preserved left ventricular systolic function (ejection fraction ~67%). Cardiac structures, including the aortic valve (AV), mitral valve (MV), interventricular septum (IVS), and left and right atria and ventricles, are well visualised. Bottom panel: Continuous-wave Doppler imaging in the apical five-chamber view. Panel (C) shows a symmetrical, non-late-peaking spectral profile with markedly elevated LVOT velocity of 4.82 m/s, consistent with subvalvular obstruction. Panel (D) demonstrates an aortic regurgitation jet with a pressure half-time (PHT) of 405 ms, consistent with mild-to-moderate central AR. AO = aorta; AV = aortic valve; MV = mitral valve; SAM = subaortic membrane; LV = left ventricle; RV = right ventricle; RA = right atrium; IVS = interventricular septum; LVPW = left ventricular posterior wall; LVOT = left ventricular outflow tract; AR = aortic regurgitation; PHT = pressure half-time

**Figure 4:**
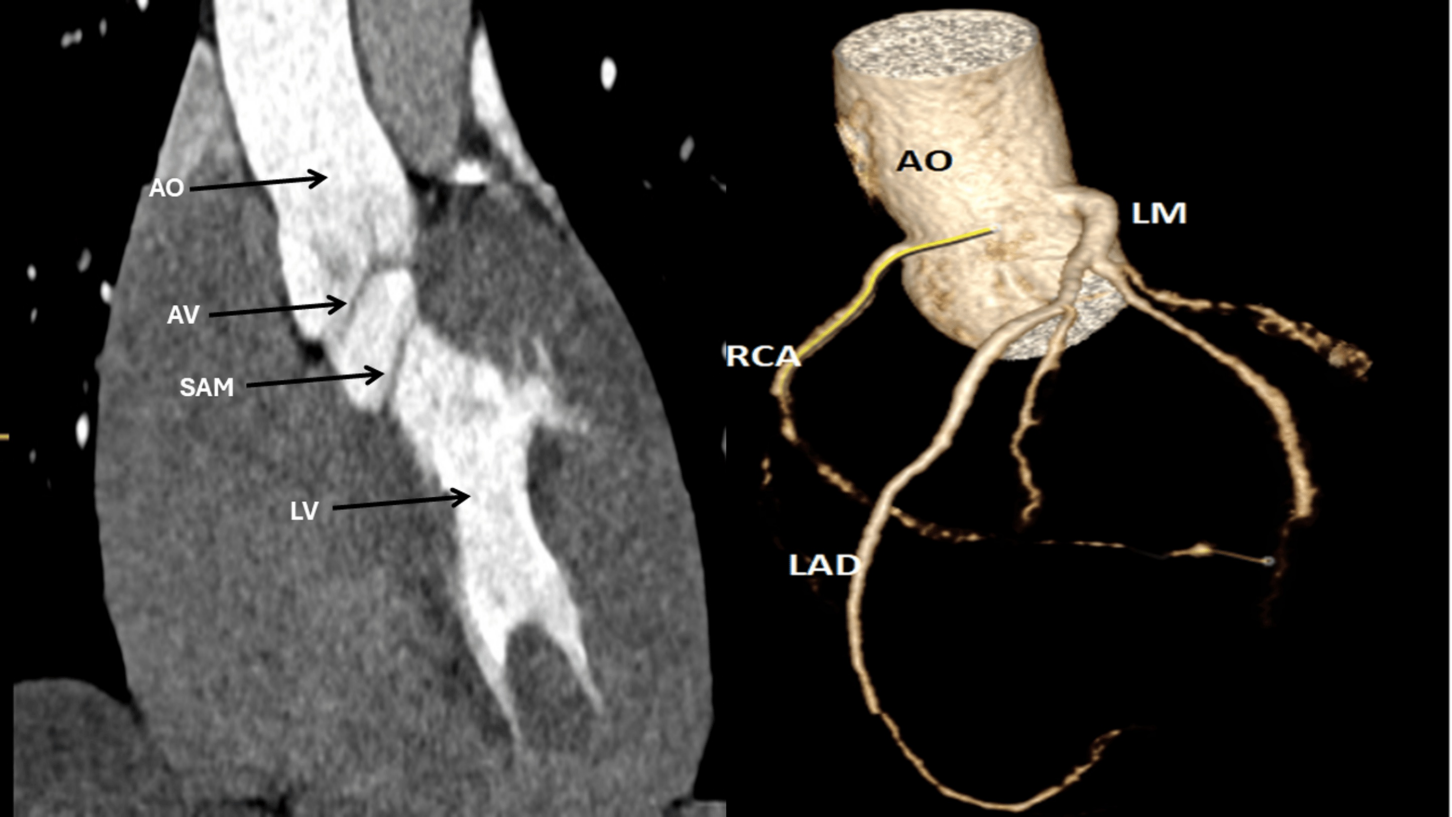
Multiplanar and 3D cardiac CT imaging demonstrating subaortic membrane and coronary anatomy Left panel: Retrospective ECG-gated contrast-enhanced cardiac computed tomography (CT) showing a reconstructed long-axis view of the left ventricular outflow tract (LVOT). The aortic valve (AV) is indicated by the red arrow, and a discrete subaortic membrane (SAM) is visualised just below the valve (black arrow). Right panel: 3D volume-rendered reconstruction of the aortic root and coronary arteries. The origins and proximal segments of the left main (LM), left anterior descending (LAD), and right coronary artery (RCA) are clearly visualised, with no evidence of coronary artery disease or anatomical anomalies. LV = left ventricle; AO = aorta; AV = aortic valve; SAM = subaortic membrane; LM = left main coronary artery; LAD = left anterior descending artery; RCA = right coronary artery

Transthoracic echocardiography (TTE) identified a tri-leaflet AV with normal systolic motion, along with a discrete subaortic membrane measuring 0.5 cm. Colour Doppler demonstrated mild to moderate central AR. Hemodynamic assessment showed a peak LVOT velocity of 4.82 m/s and a mean pressure gradient of 45 mmHg, consistent with significant subvalvular obstruction. The left ventricular ejection fraction (EF) was preserved at over 55%, and there was moderate concentric LVH, with a posterior wall thickness of 13.8 mm and a left ventricular mass index (LVMI) of 91 g/m² (Figure 3 and Videos 1-4). Cardiac CT angiography confirmed the presence of a thin, linear subaortic membrane in the LVOT, with no evidence of coronary artery disease or additional structural anomalies (Figure 4).

**Video 1:** Two-dimensional transthoracic echocardiography (parasternal long-axis view) Greyscale imaging shows a discrete subaortic membrane located in the left ventricular outflow tract (LVOT) beneath the aortic valve.

**Video 2:** Two-dimensional transthoracic echocardiography (parasternal long-axis view) Colour Doppler imaging reveals turbulent systolic flow beginning proximal to the aortic valve, consistent with a subvalvular obstruction.

**Video 3:** Two-dimensional transthoracic echocardiography (apical three-chamber view) Zoomed view of the left ventricular outflow tract (LVOT) clearly demonstrates the subaortic membrane positioned just below the aortic valve, impinging on the outflow tract.

**Video 4:** Two-dimensional transthoracic echocardiography (apical five-chamber view) Colour Doppler imaging again highlights systolic flow turbulence originating below the aortic valve, confirming dynamic obstruction related to the subaortic membrane.

Surgical decision-making and rationale

The decision to proceed with surgery was based on several key factors. While stress echocardiography is valuable for gradient escalation assessment, it was deferred in this case due to reproducible, exertion-limited symptoms and consistent imaging findings. The diagnostic yield of stress testing was considered low and unlikely to alter the management course [8].

Despite the peak gradient of 45 mmHg falling below the classic surgical threshold, the presence of NYHA Class II-III symptoms and moderate AR represents a clinically significant burden. Literature supports early intervention in symptomatic patients with gradients between 30 and 50 mmHg due to the risk of AV damage [7]. After a multidisciplinary discussion, including cardiology, cardiac surgery, and patient-family counselling, the team opted for elective surgical resection.

He underwent successful subaortic membrane excision. Cardiopulmonary bypass time was 54 minutes, with an aortic cross-clamp time of 38 minutes. No concomitant valve intervention was required, as intraoperative assessment confirmed preserved cusp morphology and only mild central regurgitation. No perioperative complications were encountered.

However, histologic confirmation was not performed, which is a limitation in precisely categorising the membrane type, whether the membrane was fibrous or fibromuscular. While this did not alter surgical management, it restricts pathophysiological insights and limits comparison with published recurrence predictors.

Transoesophageal echocardiography (TOE) confirmed complete resection (Table 1), significantly improved gradients, and no evidence of residual obstruction or ventricular septal defect (VSD). He was extubated within 24 hours and discharged home by postoperative day 7 after an uneventful recovery.

**Table 1:** Hemodynamic and structural changes before and after surgical resection of the subaortic membrane This table summarises the patient's echocardiographic findings across three key timepoints: preoperative assessment, immediate post-operative transoesophageal echocardiography (TOE), and discharge transthoracic echocardiography (TTE). The observed mild rise in left ventricular outflow tract (LVOT) velocity at discharge is likely attributable to post-extubation hemodynamic normalisation and does not indicate residual obstruction.

Parameter	Pre-operative (TTE)	Post-operative (TOE)	On discharge (TTE)
LVOT obstruction peak velocity	4.82 m/s	1.45 m/s	1.59 m/s
LVOT mean gradient	45 mmHg	4 mmHg	6.1 mmHg
Aortic valve (AV) velocity	2.73 m/s	1.68 m/s	2.11 m/s
AV mean gradient	11 mmHg	6 mmHg	9 mmHg
Subaortic membrane	0.5–0.6 cm ridge	Absent	Absent
Aortic regurgitation	Mild–Moderate (PHT 402 ms)	Minimal/none	Mild AR
Ejection fraction	>55%	~70%	60-65%
Left ventricular hypertrophy	Moderate	Improved (follow-up pending)	Increased relative wall thickness

Plan and follow-up

Our follow-up strategy includes TTE at 3 and 6 months, then annually, with escalation to 3D echocardiography if gradients exceed 20 mmHg (a value associated with subclinical recurrence in prior observational series)[1,7], and cardiac MRI at 1 year to assess for fibrotic remodelling [7]. Such structured surveillance is vital given the recurrence risk, which ranges from 20-55%, depending on anatomical and surgical factors [7]. Additionally, the patient will receive lifestyle adjustment advice, and clearance for return to sports will be considered following a stress test assessment.

At the 3-month follow-up, echocardiography showed a mean LVOT gradient of 6 mmHg and persistence of only mild central AR. The patient remained asymptomatic (NYHA I) and had resumed full physical activity. Endocarditis prophylaxis was discussed, and the patient was counselled on dental hygiene and infection risk reduction. A tailored sports clearance plan was implemented, with return to basketball permitted after a normal exercise stress test.

## Discussion

SAM represents a rare and complicated adult diagnosis, which presents in one of the following three ways: a thin, discrete ridge, fibromuscular ridge, or diffuse fibromuscular tunnel-like narrowing of the LVOT [9]. The presentation as a discrete ridge most commonly occurs, and our patient likely had it, although the diagnosis must be established by surgical resection and histopathological confirmation.

The aetiology of SAM includes genetic predisposition and mechanical forces, and the disease advances insidiously in adults to lead to progressive widening of the LVOT obstruction [9-11]. Although genetic predisposition has been proposed in isolated populations, no familial/syndromic pattern was noted in our patient. The obstruction gives rise to concentric left ventricular hypertrophy and possibly a septal bulge. The disease clinically presents as the patient transits from the asymptomatic state to developing symptomatic exertional dyspnoea, angina, palpitations, or syncope as the gradient rises [12].

Progression in adults is usually slow, while children's cases may have rapid and unpredictable progression [9]. This partly explains why our patient remained undiagnosed until adolescence, as early exertional symptoms were mild and initially attributed to deconditioning rather than structural heart disease. Such diagnostic delay is typical in adult-presenting SAM and underscores the need for heightened clinical suspicion in young patients with unexplained murmurs or LV hypertrophy.

SAM can be challenging to diagnose since it can mimic other LVOT pathologies such as hypertrophic cardiomyopathy, bicuspid AV disease, or rheumatic heart disease [9]. Transthoracic and transoesophageal echocardiography remain the standard of reference for the assessment of membrane anatomy, severity of LVOT obstruction, and AV disease [13]. Stress testing provides additional benefit in adults with ambiguous symptoms or gradients by unmasking exercise-induced obstruction or arrhythmias [13].In our patient, earlier evaluation with echocardiography may have identified the lesion before the onset of moderate AR, raising the question of whether earlier surgical referral might have altered long-term valve outcomes. Evidence from series comparing young adults with older cohorts suggests that earlier intervention may mitigate the progression of AR and reduce recurrence rates [14].

Newer applications of cardiac imaging, such as cardiac magnetic resonance (CMR) and CT, enhance echocardiography through increased anatomical definition and flow measurement [14,15]. While CMR offers high-resolution imaging and flow quantitation, visualisation of the thin subaortic membrane can be restricted by the occurrence of spin dephasing artefacts [15]. Spatial resolution of the heart in these situations may be improved using CT imaging, and it can be used as a useful adjuvant if the echocardiographic windows are poor [14].

Notably, AR severity does not correlate directly with LVOT gradient in all patients, as regurgitation is driven by turbulent jet impact and cusp trauma rather than gradient magnitude alone [9]. Disease duration and membrane proximity to the aortic cusps appear to be more predictive of AR development. Surgical management recommendations of the American Heart Association (AHA) support surgical intervention in patients presenting symptoms whose maximum LVOT gradient lies between 30-50 mmHg, as well as in asymptomatic patients whose gradients exceed 50 mmHg [13].

Surgical resection remains the definitive and most effective therapy, with high long-term outcomes, such as 20-year survival of 97% [15]. Other therapies, such as percutaneous balloon dilation, have been described but do not have sufficient long-term outcomes and are not standard recommendations [16]. Emerging therapies, including catheter-based resection or minimally invasive endoscopic techniques, remain investigational and are not currently standard of care. Their potential role in adults may expand in the next decade, particularly if adjunctive anti-fibrotic therapies are developed. Aggressive surgical strategies such as concomitant septal myectomy have been associated with lower recurrence rates in some series, though at the expense of higher operative risk [17].

One of the significant challenges of the management of SAM is the high postoperative recurrence rate, reported in certain case series to be as high as 55% [7,16], attributed to residual fibrous tissue, abnormal LVOT geometry, and inherent fibroproliferative tendencies. This underscores the importance of systematic and lifelong postoperative follow-up, traditionally through annual echocardiographic evaluation [7,16]. Current evidence recommends the use of multimodality imaging, including three-dimensional echocardiography and newer CMR modalities, to improve surgical planning and thereby reduce the need for reoperation [14].

As shown in Table 2, our approach to management lies somewhere between the conservative and more aggressive strategies described in the literature. Although the LVOT gradient did not exceed the commonly used threshold of 50 mmHg, the combination of moderate aortic regurgitation (AR) and clear clinical symptoms justified our decision to proceed with early surgical intervention. This is in line with current recommendations and supports the growing body of evidence favouring proactive surgery in symptomatic patients, even when gradients are borderline [1,7,13,16].

**Table 2:** Comparative perspectives on diagnosis, management, and follow-up strategies for subaortic membrane (SAM) in adults Comparative perspectives on the diagnosis, management, and follow-up of subaortic membrane in adults, highlighting the differences between traditional guidelines, aggressive surgical approaches, and evolving imaging-based strategies. This framework contextualises our case within the shifting paradigm toward personalised and symptom-guided management, particularly in patients with moderate gradients and coexisting aortic regurgitation. SAM: Subaortic Membrane, AR: Aortic Regurgitation, MR: Cardiac Magnetic Resonance, 4D Flow CMR: Four-Dimensional Flow Cardiac Magnetic Resonance, LVOT: Left Ventricular Outflow Tract, AI: Artificial Intelligence, 3D Echo: Three-Dimensional Echocardiography

Aspect	Our Case Report	Conservative (Guideline-Based) Approach	Aggressive Surgical Approach	Emerging / Advanced Imaging-Guided Approach
Aetiology of SAM	Adult-presenting SAM, suspected delayed manifestation of congenital origin.	Most adult cases are interpreted as late diagnoses of congenital SAM rather than newly developed lesions [2].	Aetiology is likely congenital; timing of onset is considered less critical than rate of disease progression [7].	Highlights potential for genetic predispositions and anatomical imaging parameters (e.g., aorto-septal angle, mitral-aortic distance) to differentiate true congenital vs acquired-like SAM phenotypes [10].
Threshold for Surgical Intervention	Surgery is at a mean gradient of 45 mmHg with exertional symptoms, despite not meeting classic guideline thresholds.	Recommends surgery when peak gradient ≥50 mmHg, rapid progression, or evidence of left ventricular dysfunction/symptoms [13].	Advocates early surgery if symptomatic—even with moderate gradient (30–50 mmHg)—to prevent progression [6].	Supports personalised threshold, integrating 4D flow CMR and pressure recovery dynamics to determine surgical timing [15].
Role of Aortic Regurgitation (AR)	Moderate central AR influenced the decision for surgery.	AR without a high gradient may not warrant surgery unless progression or ventricular dysfunction occurs [8].	Strongly favours early surgery when AR and obstruction coexist, to minimise aortic valve damage and remodelling [7].	Recommends quantification of AR severity using 4D flow CMR and regurgitant fraction analysis to optimise timing [16].
Management of Recurrence	Annual transthoracic echocardiography; no formal recurrence prediction model applied.	Routine post-operative echo (usually annually) without risk stratification [13].	Emphasises complete membrane resection and proper technique to reduce recurrence [6].	Proposes multimodal surveillance (3D echo + CMR) and highlights AI-driven models to identify higher-risk patients [17].
Long-Term Outcome Considerations	Acknowledges recurrence risk (20–55%) but lacks a structured strategy.	Monitors for re-obstruction; considers re-intervention if gradient >30 mmHg postoperatively [13].	Suggests early surgery may reduce the risk of multiple interventions and prevent irreversible changes [6].	Emphasises development of imaging biomarkers (e.g., LVOT turbulence, shear stress, early fibrosis) to predict recurrence/subclinical progression [11].
Use of Medical Therapy	Not addressed.	This may use beta-blockers or afterload reducers in asymptomatic/borderline patients, though the benefit is unproven [2].	Discourages medical therapy as it does not alter the disease course [8].	May consider medication as an adjunct to imaging surveillance, not primary treatment [13].
Use of Non-Invasive or Alternative Therapies	Not considered due to lack of evidence/availability.	It may be considered in non-surgical candidates (elderly/high-risk), but evidence is limited [2].	Dismisses alternatives; advocates surgery as definitive treatment [7].	Investigating catheter-based membrane ablation—experimental, early-phase [18].

In published series, aggressive approaches combining membrane resection with septal myectomy have shown lower recurrence in some paediatric cohorts, but the largest adult studies found no significant reduction in reoperation rates and a higher risk of heart block requiring a pacemaker [7]. Contemporary data suggest that age at surgery, baseline gradient, and valve-membrane distance are stronger predictors of recurrence than surgical extent, supporting a tailored, morphology-guided approach [1].

One limitation in our case was the absence of stress testing, which might have offered additional clarity by linking the patient’s symptoms more directly to the degree of obstruction. Our follow-up plan, which includes annual echocardiographic surveillance, follows standard practice. However, given the recognised risk of recurrence, future monitoring may benefit from the use of advanced imaging modalities such as 3D echocardiography or cardiac MRI [16].

This case underscores the lack of consensus in adult SAM management and the need for individualised decision-making. Future management strategies should aim to incorporate multimodal imaging, formal risk stratification tools, and possibly genetic testing to better guide intervention and follow-up planning.

Importantly, adult patients with SAM-particularly younger adults like ours-form a distinct subgroup. Their care demands a thoughtful application of guideline thresholds, combined with a holistic view of symptom burden, anatomy, and recurrence risk.

Lastly, emerging research into the genetic and developmental origins of subaortic obstruction holds promise for earlier detection and potentially novel treatment pathways [10,11,17]. However, these approaches are still in the investigational phase. Unlike paediatric cases, where management algorithms are well-established, adult SAM remains an evolving field, one that calls for tailored guidelines reflective of the unique challenges in this population.

Our case adds novel insight by illustrating the challenges of surgical timing in borderline adult cases. It supports a proactive approach in symptomatic patients with moderate gradients and AR, highlighting that delayed surgery risks further aortic cusp damage and higher recurrence. Furthermore, it reinforces the importance of structured surveillance using advanced modalities such as 3D echocardiography and 4D flow CMR. Although limited by cost and accessibility, these tools may become integral to recurrence prediction and patient-specific risk stratification in future practice.

## Conclusions

Managing SAM in adults poses distinct clinical challenges, as illustrated by our case, involving a symptomatic young adult with moderate LVOT obstruction and AR. In contrast to paediatric populations, where diagnosis and treatment pathways are well-defined, adult-specific guidelines remain sparse. Much of the current adult management is extrapolated from paediatric data, which may not fully reflect the differences in anatomy, disease progression, or recurrence risk in adult patients. While multimodal imaging and detailed non-invasive assessments enhance diagnostic accuracy, surgical resection continues to be the mainstay of treatment for symptomatic individuals or those with hemodynamically significant obstruction. Novel therapies, including genetic testing and catheter-based interventions, are still investigational and not yet validated for routine adult practice. Looking ahead, stratifying patients based on anatomical predictors of recurrence, such as the membrane’s proximity to the AV, mitral-aortic separation, and residual gradients after surgery, may help tailor both surgical planning and long-term monitoring. Patient-centred outcomes, including functional recovery, sports clearance, and quality of life, should also be systematically reported in adult SAM series, as they are key determinants of long-term prognosis and patient satisfaction.

This case underscores the need for adult-specific risk stratification tools and structured surveillance protocols that consider the high likelihood of recurrence and potential long-term complications. Follow-up should ideally incorporate advanced imaging techniques, such as 3D echocardiography or cardiac MRI, and apply individualised thresholds for considering reintervention. Continued research is essential to develop evidence-based, adult-focused management strategies that optimise outcomes and reduce the need for repeat procedures.
